# Rationale for and design of the "POSTA" study: Evaluation of neurocognitive outcomes after immediate adenotonsillectomy compared to watchful waiting in preschool children

**DOI:** 10.1186/s12887-016-0758-8

**Published:** 2017-02-02

**Authors:** Karen A. Waters, Jasneek Chawla, Margaret-Anne Harris, Carolyn Dakin, Helen Heussler, Robert Black, Alan Cheng, Hannah Burns, John D. Kennedy, Kurt Lushington

**Affiliations:** 1 The Children’s Hospital at Westmead, Sydney, NSW 2145 Australia; 2 The University of Sydney, Sydney, Australia; 3 The Lady Cilento Children’s Hospital, Brisbane, Australia; 4 The Mater Research Institute - The University of Queensland, Queensland, Australia; 5 Women and Children’s Hospital, Adelaide, Australia; 6 The University of South Australia, Adelaide, Australia

**Keywords:** Intelligence, Neurocognition, Pre-school, Child, Sleep disordered breathing, Tonsillectomy, Adenotonsillectomy, Clinical trial

## Abstract

**Background:**

IQ deficits are linked to even mild obstructive sleep apnoea (OSA) in children. Although OSA is commonly first diagnosed in the pre-school age group, a randomised trial is still needed to assess IQ outcomes after adenotonsillectomy in the pre-school age-group. This randomised control trial (RCT) will primarily determine whether adenotonsillectomy improves IQ compared to no adenotonsillectomy after 12 months, in preschool (3–5 year-old) children with mild to moderate OSA.

**Methods:**

This protocol is for an ongoing multi-centred RCT with a recruitment target of 210 subjects (105 in each arm). Children age 3–5 years with symptoms of OSA, are recruited through doctor referral, at the point of referral to the Ear Nose and Throat (ENT) services. Screening is initially with a questionnaire (Paediatric Sleep Questionnaire, PSQ) for symptoms of obstructive sleep apnoea (OSA). Where questionnaires are positive (suggestive of OSA) and ENT surgeons recommend them for adenotonsillectomy, they are invited to participate in POSTA. Baseline testing includes neurocognitive testing (IQ and psychometric evaluation with the neuropsychologist blinded to randomisation) and overnight polysomnography (PSG). Where the Obstructive Apnoea-Hypopnea Index (OAHI) from the PSG is <10/h per hour, consent for randomisation is sought; children with severe OSA (OAHI ≥ 10/h) are sent for immediate treatment and excluded from the study. After consent is obtained, participants are randomised to early surgery (within 2 months) or to surgery after a usual wait time of 12 months. Follow-up studies include repeat neurocognitive testing and PSG at 12 (with the waiting list group studied before their surgery) and 24 months after randomisation. Analysis will be by intention to treat. The primary outcome is IQ at 12 months’ follow-up.

**Discussion:**

If IQ deficits associated with OSA are reversible 12 months after adenotonsillectomy compared to controls, future clinical practice advise would be to undertake early surgery in young children with OSA. The study could provide data on whether a window of opportunity exists for reversing IQ deficits linked to OSA in the pre-school age-group.

**Trial registration:**

Australian and New Zealand Clinical Trials Registration Number ACTRN12611000021976.

## Background

Children with upper airway obstruction in sleep (obstructive sleep apnea, or OSA) may display repetitive, discrete episodes of airway obstruction associated with intermittent desaturation and arousal. However, they frequently also demonstrate long periods of partial airway obstruction associated with snoring, increased work of breathing and normal gas exchange in addition to the discrete events, or as their only expression of the disease.

Snoring reportedly affects 8–12% of children while 1–3% have OSA with discrete respiratory events [1]. Thus, even at its lowest prevalence, around 1 child in every primary school classroom has OSA, equating to 80,000 affected children in Australia and 16,000 children in New Zealand [2]. Although snoring and OSA are common across the childhood age-range, studies specific to the pre-school age group are vital. The first presentation of OSA is often in this age group, when the lymphoid tissue of the adenoids and tonsils is at its largest relative to the bony skeleton of the airway, thus compromising the upper airway size [3]. Adenotonsillar enlargement is the commonest treatable element of OSA in children and adenotonsillectomy (AT) constitutes the primary treatment intervention.

Polysomnography (PSG) is the standard investigation used to detect the presence and severity of OSA [4]. PSG studies provide an audio-visual assessment of obstruction and a documentation of the frequency of apneic events; defined as the number of obstructive apnoea & hypopnea events that occur per hour of sleep time. PSG outcomes thus provide the index of events per hour (OAHI), the severity of blood gas disturbances (hypoxia and hypercapnia), and the frequency of arousals. In the pediatric literature the severity of airway obstruction is then classified from PSG as on the basis of the obstructive apnoea-hypopnea index (OAHI).

### Neurocognitive associations with OSA

Recent studies show that snoring or mild OSA are associated cognitive and behavioral deficits in school-age children [5–14]. The mechanism of the association is not clear, and studies have examined associations with hypoxia, sleep fragmentation, and lack of cerebral blood flow adaptation as likely candidates [15–18]. Most human studies pertaining to recovery of neurocognitive function examine sequelae of traumatic or acute brain injury. Importantly, a number of those studies show that younger children generally have less capacity to compensate for brain injury than older children [19]. In contrast, some small studies suggest greater potential for recovery of specific neurocognitive processes in younger children [20].

To date, a substantial amount of the literature pertaining to neurocognition in children with OSA has come from studies in school-age children. The biggest cognitive impact of OSA is on children’s executive function. The development of the components of executive function is rapid in the age-range of 3–8 years, but continues through childhood and adolescence and is aligned with growth spurts and myelination in the frontal lobes [21]. Specific links have been suggested amongst sleep, behavioral problems, and executive function because of the prolonged developmental time frame of executive function in childhood, including the potential for there being a critical period of development [22]. Limited knowledge regarding the effects of age at the time of brain injury on subsequent IQ recovery means that results from studies of OSA in school-age children cannot be confidently extrapolated into the pre-school age group. Therefore, pre-school children are a vitally important group in which to investigate the adverse effects of OSA.

### Changes after treatment of OSA

The Childhood Adenotonsillectomy trial (CHAT) is a published randomized controlled trial in children evaluating IQ outcomes after adenotonsillectomy. Children in that study were school-age aged 5–9 years (*n* = 464), with 7 month follow up post intervention [23]. That study found no effect of early AT on attention or executive function as measured by neuropsychological testing. However the positive impacts of surgery included reduced symptoms and improved behavior, quality of life and PSG findings, so the authors concluded that the study provided evidence of the beneficial effects of early AT [23]. An editorial accompanying the publication of the CHAT study suggested that studies now need to focus on children at a younger age and with longer follow-up to evaluate whether IQ deficits are at all reversible [24].

## Aims

The POSTA (Pre-school OSA Tonsillectomy Adenoidectomy study) is a randomized, controlled trial of adenotonsillectomy in pre-school age children, with mild to moderate OSA, designed to evaluate neurocognitive outcomes after early adenotonsillectomy compared to watchful waiting, with a 12-month follow-up. Subjects will be assessed at baseline and again 12 months after randomization. Subjects randomized to the control group will have surgery following their 12 month follow-up assessment (approximating the average delay to surgery in Australia for children with this severity of OSA). Subjects randomized to intervention will have AT within 2 months of randomization. The specific aim is to determine whether AT improves IQ in preschool (3.0–5.0 year-old) children with mild to moderate OSA in a group who undergo early AT compared to those with no AT after 12 months. The design will allow us to test the principal hypothesis that IQ benefits will be demonstrable 12 months after surgery as opposed to no surgery in the pre-school age-group. Table 1 shows the primary and secondary outcomes to be measured.

**Table 1:** Primary and secondary outcomes

Primary Outcome: Intellectual ability (IQ) as determined by the Woodcock-Johnson III (WJ-III) Test of Cognitive Abilities	
Secondary Outcomes:	
• Behavioral parameters (PRS-BASC-II)	
• Executive Function (BRIEF-P)	
• Parental Stress (Parental Stress Index)	
• Polysomnography (PSG) parameters	
• Sleep Symptoms (Pediatric Sleep Questionnaire- PSQ)	
• Anthropometry: change in height, weight, and BMI percentiles	
• Blood Pressure: Mean arterial pressure	
• Audiology	
• Quality of life	

## Design

### Overview of the study design

Pre-school age children (3.0–5.0 year-old) with a history of snoring will be contacted at the point of referral. The Pediatric Sleep Questionnaire (PSQ) will be used to screen for symptoms of OSA [25]. Children with a questionnaire score suggestive of OSA will be eligible for overnight sleep studies to classify the severity of their disease. Those with mild to moderate OSA (obstructive respiratory event index <10 per hour) are eligible for inclusion. Those with severe OSA (10 per hour) will be sent for immediate treatment and excluded. Recruited subjects will proceed to neurocognitive (IQ) testing and will then be randomized to either immediate or routine surgical waiting times. The neurocognitive tests (see details below) will apply to all participating children at baseline, then at 12- and 24-month follow-up. A routine surgical wait time for adenotonsillectomy means surgery 12 months after randomization, while early surgery will occur within 2 months of randomization. All children will have repeat neurocognitive testing 12 months after their randomization.

#### Recruitment strategy

Families referred to the hospital for adenotonsillectomy because of a history suggestive of OSA will be approached by mail for participation in this study. A letter, including the Pediatric Sleep Questionnaire (“PSQ”) questionnaire for OSA [25], will be sent to the families with a postage paid return envelope. Children with positive answers to ≥33% of the 22 question-items are considered to have a questionnaire diagnosis of OSA and will be invited to undergo overnight polysomnography (*PSG, see below for details*) to define the severity of their OSA. Questionnaire screening will ensure that children having PSG have high probability of OSA as this questionnaire has specificity of 0.87 for OSA in a general pediatric setting. After PSG children with an OAHI < 10 events/h will proceed to baseline testing while those with an OAHI >10 will require early clinical intervention and exclusion from further study.

#### Exclusion criteria

Children with medical conditions known to affect neurocognitive development (including hearing loss of >35 DB), previous tonsillectomy or airway surgery, high-risk genetic and craniofacial syndromes, identified neurodevelopmental conditions, cyanotic heart disease or previous cardiopulmonary bypass, and conditions that preclude neurocognitive testing or that preclude adenotonsillectomy will be excluded. Children with severe OSA (OAHI >10/h on sleep study) will also be excluded because they need to proceed to treatment to avoid morbidity from ongoing OSA. This group also has higher post-operative complication rates [26, 27]. Table 2 shows the list of inclusion and exclusion criteria.

**Table 2:** POSTA eligibility and exclusion criteria

Inclusion Criteria:	
1. Children aged 3–5 years at the time of primary assessment for evaluation of snoring	
2. Deemed suitable for adenotonsillectomy on surgical evaluation	
3. Confirmed on PSG to have mild-moderate OSA, defined as OAHI < 10 events/h	
Exclusion Criteria:	
1. Medical conditions known to affect neurocognitive development (including hearing loss >35DB)	
2. Previous tonsillectomy or airway surgery	
3. High risk genetic and craniofacial syndromes	
4. Previously identified neurodevelopmental conditions	
5. Cyanotic heart disease or previous cardiopulmonary bypass	
6. Conditions that preclude neurocognitive testing or that preclude adenotonsillectomy	
7. Confirmed on PSG to have severe OSA, define as OAHI >10/h	

*PSG* polysomnography, *OAHI* obstructive apnoea hyponoea index

#### Testing

Neurobehavioural (IQ) assessment after a hearing test (see exclusion criteria), PSG and medical review will be undertaken at baseline prior to randomization and repeated 12 months after randomization. Baseline testing also includes standardized history and medical examination and behavioral testing. The medical review will include family and personal histories of atopy, visual grading of tonsil size, measurement of body weight, height and calculated body mass index (BMI), and evening and morning blood pressure.

#### Method of randomization

After completing initial questionnaire, sleep study and IQ assessments, children will be centrally randomized either to immediate surgery or to remain as waiting list controls. Study center and severity of OSA may all influence study outcomes. To avoid imbalance between the groups, these prognostic factors will be used for stratified randomization with OSA classified as < 5 events/h or > 5 events/h by center. To avoid small cell sizes we will implement a minimization algorithm to ensure that randomization groups are balanced for the stratifying variables. Centralized randomization is through the Children’s Hospital at Westmead. Each patient will be assigned a unique trial number that will be used for recording all their data. Protocols will be standardized between centers, including sleep study analysis, IQ testing, and surgical procedures.

*Intervention* will comprise adenotonsillectomy within 2 months of the assignment date. All children will undergo complete extra-capsular tonsillectomy and adenoidectomy, regardless of the size of the adenoids. The adenoidal bed will be visualized at the end of the procedure, to ensure the adenoidectomy is satisfactorily complete. It has been agreed that diathermy for adenoids is acceptable.

The main retention strategy will be 2-monthly phone contact with participant families, during which 5 questions will be asked about the child’s health.

### Setting

The group designing the study included Paediatric Sleep Physicians and ENT surgeons representing all major Australian Paediatric centres. The study is taking place in three Australian Children’s hospitals and will take subjects from their referring centres.

#### Sample size and power of the study

The standard deviation of IQ over 1 year is estimated to be approximately 8 points [28, 29]. The study aims to detect a difference in change in IQ of at least 4 points (0.5 standard deviation units)1 year after the baseline assessment. To detect this difference, with a power on 0.9 and a significance of 0.05 we will require 86 children in each group or 172 in total.

In order to retain the intended power of the study, the sample size will need to be increased to 94 in each group or 188 in total (estimated by Pocock method in Power analysis and sample size (PASS) Statistical Software, Utah 2008). Assuming that 90% of randomised subjects have their IQ assessed at follow-up, we plan to randomise 210 subjects, that is, 105 in each group.

#### Recruitment

The final recruitment target is 210. We have been able to recruit 22 children in one centre over a 12 month period and estimate that three study centres will complete recruitment in 2.5 years, and follow-up all subjects in 3.5 years. This rate of recruitment will require that approximately one child per month have immediate surgery. During the period while recruitment and follow-up are occurring concurrently, the requirement is for 4 PSG studies per month at each centre (2 new and 2 follow-up if all of the new recruits in any 1 month are eligible).

## Specific assessment procedures

### Neurobehavioural and neurocognitive testing

A qualified psychologist will conduct neurobehavioural and neurocognitive testing on each child at each assessment. The major outcome measure will be intellectual ability as determined by the Woodcock-Johnson III (WJ-III) Test of Cognitive Abilities [30]. Measurement bias will be minimized by ensuring that the psychologists conducting the psychological assessments at follow-up are blinded to treatment group allocation. Participants will be coached to avoid disclosure.

Intellectual ability will be estimated by administering the seven core subtests of the WJ III: Verbal Comprehension, Visual-Auditory Learning, Spatial Relations, Sound Blending, Concept Formation, Visual Matching and Numbers Reversed. The composite of these subtests is the measure of IQ, termed GIA in the WJ III. Amongst candidate IQ tests, the WJ-III is unique in permitting testing over the proposed age range (i.e., 3.0–6.9y inclusive) using the same test items at different age bands. In addition to estimating general intellectual ability, the core subtests of the WJ III can be used to examine more specific cognitive skills that may be sensitive to sleep disruption. In the 3–6 y age range, specific cognitive abilities measured by the core subtests include: language development and verbal knowledge, complex thinking processes (long term retrieval of information, visual-spatial thinking, auditory processing and fluid reasoning), and more automatic thinking processes (short term memory and processing speed). Two additional WJ III subtests will be used to provide further assessment of attention and executive function: Retrieval Fluency and Picture Recognition. The WJ-III is normed on a sample of >8100 US children and is reported to have high test-retest reliability, high internal consistency, strong convergent validity and a robust factor structure [30]. WJ-III testing time is estimated to be 30–50 min.

*Behavioral parameters* and fluid skills incorporated in executive function are deleteriously affected by OSA. These will be further tested using the Parent Rating Scale of the Behavioral Assessment System for Children-II (BASC-PRS) [31] and the Behavior Rating of Executive Functioning (BRIEF-P) [28].

The BASC-PRS contains 160 items and uses a four-choice response format. In the 3-6y age range, the BASC-PRS provides information about a range of areas of functioning including emotional and psychological functioning (e.g., anxiety, depression), behavior (e.g., aggression, activity levels), social skills and interaction, attention and activities of daily living. It is reported to possess high test-retest reliability, high internal consistency and a robust factor structure [31]. The BRIEF-P consists of 63 items and uses a three-choice response format and provides information about executive function in everyday settings including skills such as behavioral and emotional control, planning and organization, attention and information processing. It is reported to possess high internal consistency and reliability, good convergent and discriminant validity and a robust factor structure [28]. The PRS is estimated to take 15 min and the Brief-P 15 min for parents/caregivers to complete.

Finally, parent stress is associated with sleep and behavior difficulties in children so this will be assessed using the Parenting stress Index (PSI) [32]. The PSI consists of 120 questions and yields a Total Stress Score, a Child Domain score, a Parent Domain score and a Life Stress score. Child characteristics are measured on 6 subscales: Distractibility, Hyperactivity, Reinforces Parent, Demandingness, Mood and Acceptability. Parent personality and situational variables are measured on 7 subscales: Competence, Isolation, Attachment, Health, Role Restriction, Depression and Relationship with Spouse. The PSI has satisfactory internal consistency, robust test-retest reliability [32]. The PSI takes 20–30 min to complete.

The maximum testing time (including breaks) for neurobehavioural and neurocognitive testing is estimated to be 2 h, but 4 h will be allowed for each child for the full testing battery.

#### Polysomnography (PSG)

Each participant identified by questionnaire will undergo full overnight sleep studies using standard clinical pediatric techniques and analyses. At each acquisition point, the sleep study results will only be included if they capture a minimum of 4 h recording time and at least 2 cycles of REM sleep; if these are not obtained, a repeat PSG will be undertaken.

Total sleep time and the obstructive apnoea hypopnea index (AHI) defined as the total number of obstructive apnoeas, mixed apnoeas, and obstructive hypopneas per hour of sleep will be calculated. PSG will be scored using American Academy of Sleep Medicine (AASM) 2007 guidelines [33], as these guidelines were current at the time of study design and its commencement.

#### Sleep study concordance

As sleep studies will be carried out in 3 different sleep laboratories, a maximum of two experienced sleep technologists will be solely responsible for analysis of the PSG studies in each center. Analysis criteria have been agreed upon and those involved in analyzing studies will meet regularly to evaluate studies and ensure concordance. No published data exists on multi-center trial concordance for pediatrics.

The severity of OSA is defined by the number of obstructive apnoea & hypopnea events that occur per hour of sleep time expressed as the index of events per hour (OAHI). Standard diagnostic categories for pediatric OSA will be used and are: Primary Snoring <1 event/h; mild OSA 1–5 events/h; moderate OSA 5–10 events/h; and severe OSA >10 events/h [34]. Severe OSA experience nocturnal respiratory failure and severe desaturations, and there is clinical imperative for them to receive the beneficial effects of adenotonsillectomy [34–36].

#### Data collection & storage

This study will generate a significant amount of data, due to the large amounts of data points per test and the 2 time points of study. The database for POSTA has been custom-designed to allow handling of this data. It is a secure centralized web-based system that allows transfer of data directly from source for many of the data points. Each study site can upload information directly into the centralized system, ensuring clean and compatible data for analysis. The direct export of PSG and neurocognitive data into this central database will minimize errors that could otherwise occur from manual data entry. Summary data can be generated and exported to relevant statistical packages according to requirements for data analysis (http://www.postastudy.org).

## Discussion

### Management of recruitment challenges

Acknowledged challenges of clinical trials include parental (participant) perceptions of a randomised treatment, including surgical procedures [37]. Our protocol, requiring repeat sleep studies and assessments, is challenging for young families where younger siblings are common, and this may delay recruitment or lead to high dropout rates. The protocol has been standardised across sites, but some disparities in practice will inevitably evolve across different sites. For example, in Sydney children with uncomplicated adenotonsillectomy ATs are increasingly likely to have surgery performed in secondary, rather than tertiary hospitals. Informing the relevant departments and staff, and obtaining ethical approval for recruitment from these sites can take considerable time and families from those locations have considerable additional travel requirements if they choose to participate because paediatric sleep facilities are only available at the tertiary centres. Disruption to the protocol also occurred when one hospital move to new facilities, a factor which may occur again.

The primary outcome of follow-up at 12 months is our priority and we are confident we can get follow-up of 180 children (90 in each arm) with current funding.

#### Outcomes and significance

As demonstrated in the background literature, the clinical questions being addressed by this study are of national and international significance. Large, prospective randomized trials are required if we are to measure, with accuracy, whether early deficits in IQ resulting from OSA are reversible in the pre-school age-group. This study will also help determine whether the timing of adenotonsillectomy needs to be changed in pre-school children with mild to moderate OSA.
